# Discovery of Novel Thanatin-like Antimicrobial Peptides from Bean Bug *Riptortus pedestris*

**DOI:** 10.3390/pharmaceutics16111453

**Published:** 2024-11-14

**Authors:** Pavel V. Panteleev, Julia S. Teplovodskaya, Anastasia D. Utkina, Anastasia A. Smolina, Roman N. Kruglikov, Victoria N. Safronova, Ilia A. Bolosov, Olga V. Korobova, Alexander I. Borzilov, Tatiana V. Ovchinnikova

**Affiliations:** 1 M.M. Shemyakin & Yu.A. Ovchinnikov Institute of Bioorganic Chemistry, Russian Academy of Sciences, 117997 Moscow, Russia; juliyateplovodskaya@gmail.com (J.S.T.); nastin-u@yandex.ru (A.D.U.); a.a.smolina003@mail.ru (A.A.S.); kruglikov1911@mail.ru (R.N.K.); victoria.saf@ibch.ru (V.N.S.); bolosov@ibch.ru (I.A.B.); ovch@ibch.ru (T.V.O.); 2 Moscow Center for Advanced Studies, 123592 Moscow, Russia; 3 State Research Center for Applied Microbiology & Biotechnology (SRCAMB), 142279 Obolensk, Russia; korobova@obolensk.org (O.V.K.); borzilov@obolensk.org (A.I.B.)

**Keywords:** antimicrobial peptide, thanatin, *Riptortus pedestris*, transcriptome mining, in vivo efficacy, LptA

## Abstract

**Background:** Endogenous antimicrobial peptides (AMPs) are evolutionarily ancient molecular factors of innate immunity that play a key role in host defense. The study of the diversity of animal defense peptides has important applications in the context of the growing global antimicrobial resistance. **Methods:** In this study using a transcriptome mining approach, we found three novel thanatin-like β-hairpin AMPs in the bean bug *Riptortus pedestris*, named Rip-2, Rip-3, and Rip-4. The peptides were expressed in the bacterial system, and their antimicrobial activities were evaluated both in vitro and in vivo. **Results:** Homologs of the discovered AMPs are widely distributed among different members of the infraorder Pentatomomorpha. Rip-2 was shown to have the most similar structure and LptA-targeting mechanism of action to those of thanatin, but the former peptides demonstrated a higher activity against key Gram-negative ESKAPE pathogens and also displayed a significant efficacy in a lethal model of septicemia caused by *E. coli* in mice at daily doses greater than 5 mg/kg. In contrast, Rip-3 and Rip-4 peptides caused bacterial membrane damage, did not induce bacterial resistance, and exhibited a strong selectivity against *Bacillus* and *Mycobacterium* spp. **Conclusions:** This study extends the knowledge of the structure and functions of insect host defense AMPs. Each of the novel β-hairpin peptides has a potential to be a template for the development of selective antibiotic drugs.

## 1. Introduction

Endogenous antimicrobial peptides (AMPs) are among the earliest molecular factors in the evolution of innate immunity. They are essential for host defense in most multicellular organisms. The molecular characterization of AMPs is essential for the understanding of symbiont-host cross-talk. In addition, the study of the diversity of animal defense peptides has important applications in the context of the growing global antimicrobial resistance. Not only more active isoforms of known AMPs, but also fundamentally new peptide scaffolds with antimicrobial activities can be discovered by an approach based on a deep analysis of animal defense peptide repertoires. The bioinformatic search for new AMPs can be based on the homology of various structural elements of corresponding precursor proteins: signal sequences [1], conserved domains [2], or structural elements of mature AMPs [3].

The Heteroptera bugs are of great interest in terms of the search for new AMPs. To date, approximately 40,000 species of this suborder have been described, many of which were characterized by published transcriptome and genome sequencing data, facilitating bioinformatic searches. These bugs are ubiquitous and use different classes of host defense peptides in their arsenal; for example, the bean bug *Riptortus pedestris* has been found to have rip-thanatins, defensin, the proline-rich AMP riptocin, and crypt-specific cysteine-rich peptides (CCRs) [4,5]. The most studied AMP from Heteroptera is thanatin, which was first isolated from the spined soldier bug *Podisus maculiventris* in 1996 [6]. The peptide contains 21 amino acids, with the disulfide bond between Cys11 and Cys18, and has a selective mechanism of action unusual for β-hairpin AMPs. Thanatin disrupts the bridge between the inner membrane proteins LptBFG and LptD by binding to the LptA protein in the periplasmic space. As a result, this inhibits lipopolysaccharide (LPS) transport and outer membrane biogenesis in some Enterobacteriaceae [7]. In addition, the direct interaction of thanatin with LPS is thought to be a key step in the permeabilization of bacterial outer membranes [8]. Thanatin-like peptides could be attractive scaffolds for the development of less expensive antibiotics compared to AMPs, which are stabilized by multiple disulfide bonds, such as defensins. However, the relatively narrow spectrum of activities of known natural thanatins and a high probability of antimicrobial resistance necessitate the development of modified variants [9], as well as further search for homologs [10] bearing key structural elements of these molecules. 

In this work, genes encoding thanatin-like AMPs were found in genomes and transcriptomes of Heteroptera bugs. Detailed analysis of these genes and corresponding protein sequences from the bean bug *R. pedestris* revealed three new β-hairpin AMPs of variable length, ranging from 18 to 25 amino acid residues, characterized by a distinct antibacterial spectrum and mechanism of action.

## 2. Materials and Methods

### 2.1. Identification and Recombinant Production of New Rip-Thanatins

The TBLASTN software (https://blast.ncbi.nlm.nih.gov/Blast.cgi, last accessed 10 June 2024) was used to identify AMP genes in the Transcriptome Shotgun Assembly (TSA) and whole-genome sequencing (WGS) GenBank databases deposited for Heteroptera species using precursor proteins of known rip-thanatin [4] or novel peptides from *R. pedestris* as a query with the values of the default parameters (matrix: BLOSUM62, gap costs: existence 11, extension 1). Then, the obtained hit sequences were manually analyzed to identify fragments encoding mature AMPs. The corresponding genes optimized to be expressed in *E. coli* were generated by the annealing of partially self-complementary oligonucleotides (Table S1), followed by PCR and cloning in the pET-based vector as described previously [2]. Transformed *E. coli* BL21 (DE3) cells were grown at 37 °C to OD_600_ 1.0 in lysogeny broth (LB) medium containing 20 mM glucose, 100 μg/mL of ampicillin, and 1 mM MgSO_4_, and then were induced with 0.3 mM of isopropyl β-D-1-thiogalactopyranoside (IPTG, Sigma, St. Louis, MO, USA). The induction was performed for 16 h at 30 °C and at a shaking speed of 220 rpm. Purification of the target peptides was performed by immobilized metal affinity chromatography (IMAC), BrCN cleavage of the fusion protein, and reverse-phase high performance liquid chromatography (RP-HPLC) as described previously [11]. To allow BrCN cleavage of the fusion protein with thanatin, the Met21 residue in its structure was also replaced with leucine. RP-HPLC peaks collected by monitoring at 214 and 280 nm were analyzed by MALDI-TOF mass-spectrometry (Bruker Daltonics, Bremen, Germany). The obtained fractions with confirmed molecular masses (Table S1) were dried and dissolved in water. For animal experiments, peptides Rip-2 and thanatin [M21L] (hereinafter, “thanatin”) were produced in endotoxin-free ClearColi BL21(DE3) (Lucigen) expression system using the same protocol. The other peptides used were previously obtained in our laboratory (Table S2).

### 2.2. Circular Dichroism Spectroscopy

Secondary structures of the target peptides were studied by circular dichroism (CD) spectroscopy with the use of a Jasco J-810 instrument (Jasco, Tokyo, Japan). Experiments were performed at 25 °C in water, in 30 mM of dodecylphosphocholine (DPC, Anatrace, Maumee, OH, USA) micelles, or in 30 mM of sodium dodecyl sulfate (SDS, Sigma, St. Louis, MO, USA) micelles. The final peptide concentration was 300 µM.

### 2.3. Antimicrobial Assay

Bacteria were grown to mid-log phase at 37 °C in the Mueller-Hinton broth (MHB, Sigma) and then diluted with the same 2× medium supplemented with 1.8% NaCl to a final cell concentration of 10^6^ colony forming units (CFUs) per mL. A total of 50 μL of the resulting cell suspension was added to aliquots of 50 μL of peptide solutions serially diluted with 0.1% bovine serum albumin in 96-well flat-bottomed polystyrene microplates (Eppendorf, Hamburg, Germany). After 24 h incubation at 37 °C and shaking at 900 rpm, the minimum inhibitory concentrations (MICs) were calculated. The Middlebrook 7H9 medium (HiMedia, Mumbai, India) was used to determine the MICs of the peptides against *Mycobacterium smegmatis*. The results were expressed as the median values of three independent experiments performed in triplicate. The detailed characteristics of utilized bacterial strains and clinical isolates are presented in [2,11].

### 2.4. Selection of Peptide-Resistant E. coli and Whole-Genome Sequencing

Serial passage resistance induction by Rip-2 (4 independent experiments), Rip-3, and Rip-4 against the sensitive clinical isolate *E. coli* MDR 1057 (SRCAMB collection № B-10910) was performed as described previously [2]. For each subsequent daily transfer, the inoculum (2 μL) picked from the first well containing a subinhibitory concentration of the peptide were diluted 500-fold with the fresh 2× medium supplemented with 1.8% NaCl. Next, 50 µL of the resulting suspension was sub-cultured into the next passage wells containing 50 µL of the peptide solutions at concentrations ranging from 0.25× to 8–16× of the current MIC of each compound. Bacteria growing in the presence of the highest concentrations of AMPs on day six were passaged on agar plates without peptides for three days before the final MICs were determined. Next, 2 × 100 bp pair-end sequencing of genomic DNA was performed with an Illumina NextSeq550 platform (Illumina, San Diego, CA, USA). The obtained sequencing data analysis was performed as described previously [12]. The genome of wild-type *E. coli* MDR 1057 strain was used as a reference [12]. To prove the obtained data, the analyzed genes were amplified by PCR using specific primers (Table S1) and inserted into the pAL-2T vector (Evrogen, Moscow, Russia), followed by Sanger sequencing using the ABI PRISM 3100-Avant automatic sequencer (Applied Biosystems, Foster City, CA, USA).

### 2.5. Bacterial Membranes Permeability Assay

To test the ability of the peptides to damage the cytoplasmic membrane of Gram-negative bacteria, an assay with the use of *E. coli* ML-35p strain and o-nitrophenyl-β-D-galactoside (ONPG, AppliChem, Darmstadt, Germany) was performed in 96-well flat-bottom microplates [2]. The washed bacterial cells (5 × 10^6^ CFUs in each well) in phosphate-buffered saline were mixed with the test peptides and 2.5 mM ONPG. The final volume in each well of the plate was 0.2 mL. The experiment was performed at 37 °C under stirring at 300 rpm. Absorbance of o-nitrophenol was monitored at 405 nm. Three independent experiments were performed, and the curve pattern was similar for all three series.

### 2.6. Hemolytic and Cytotoxic Activities

The hemolytic activity of the peptides was tested using the fresh suspension of human red blood cells (hRBCs) by the hemoglobin release assay [2]. Using hRBCs from independent donors, two experiments were performed. Cytotoxicity of the peptides was tested on human peripheral blood mononuclear cells (PBMCs) using the resazurin assay [1]. Briefly, 50 μL of the cell suspension (10^5^ cells per well) in RPMI-1640 supplemented with 10% FBS (Invitrogen, Waltham, MA, USA) were added to aliquots of 50 μL of the peptide solutions serially diluted with the same medium in 96-well flat-bottom polystyrene microplates. After incubation for 20 h in the CO_2_-incubator (5% CO_2_, 37 °C), 5 µL of resazurin water solution (a final concentration of 0.1 mg/mL) was added to each well, followed by additional incubation for 4 h. Resorufin fluorescence was registered using a 535/595 filter at microplate reader AF2200 (Eppendorf, Hamburg, Germany). The experimental data were obtained from two independent experiments performed in triplicate. The data are presented as average means ± SD.

### 2.7. Systemic Septicemia Infection Mice Model

Female 8–10-week-old BALB/c mice (22–24 g) were infected with 500 µL of bacterial suspension of *E. coli* ATCC 25922 (10^6^ CFUs per animal) in saline containing 2.5% mucin (*w*/*v*) via intraperitoneal (IP) injection as described previously [2]. A total of seven groups of five animals each were used. The first group received saline (as a vehicle control) administered via IP once (1 h post-infection). The second group received ciprofloxacin (Sigma, St. Louis, MO, USA) administered via IP twice (1 h and 4 h post-infection) at a dose of 10 mg/kg each time. The other 5 groups received the tested peptides in different doses administered via IP twice (1 h and 4 h post-infection). After the 7-day experiment, the survived animals were euthanized by CO_2_ asphyxiation. The spleens were removed aseptically, homogenized, serially diluted, and plated on Endo agar for the determination of CFUs.

## 3. Results and Discussion

### 3.1. Novel Thanatin-like Peptide Subfamilies Were Found in the R. pedestris Genome and Transcriptome

**Figure 1:**
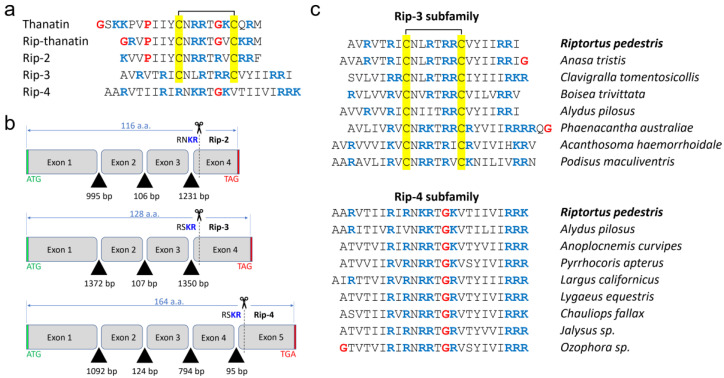
Analysis of new thanatin-like peptides identified in the bean bug *Riptortus pedestris*. (**a**) Amino acid sequence alignment of the discovered peptides with known AMPs. (**b**) Gene structures of Rip-2, Rip-3, and Rip-4 deduced by alignment of found transcripts with the reference genome of *R. pedestris* (WGS project JADPXZ01). A detailed gene analysis is presented in Figure S1. (**c**) Homologs of Rip-3 and Rip-4 are widespread among different representatives of the infraorder Pentatomomorpha.

The Rip-2 and Rip-3 peptide sequences were detected in the assembled transcriptome of *R. pedestris* (GenBank: GKOW00000000.1) [5] by the TBLASTN algorithm and by using different structural elements of the precursor protein of the previously discovered rip-thanatin as a query. In particular, we analyzed all reading frames of the translated transcriptome for the presence of the relatively conserved cysteine loop pattern C-X_6_-C, which is characteristic of the thanatin family [10]. Notably, both found peptides lack the conserved glycine residue in the loop (Figure 1a). In addition, the Rip-3 peptide is characterized by an extra *C*-terminal piece. Surprisingly, further mining of the *R. pedestris* transcriptome using the Rip-3 precursor protein sequence (Figure S1) as a query led to the discovery of another homologous peptide named Rip-4 and lacking conserved cysteine residues in the loop. 

All three precursor proteins contain *N*-terminal signal sequences, the acidic prodomain (from 74 to 114 aa) followed by the putative furin cleavage site Lys-Arg, and a *C*-terminal AMP part (18, 22, and 25 aa) enriched in positively charged residues (Figure S1). Despite the similarity of the mature AMPs, the Rip-2 and Rip-3 genes do not exhibit significant homology and only share a common architecture—the number of exons/introns and their size (Figure 1b). The Rip-4 peptide gene has five exons and no significant homology with other *R. pedestris* AMP-coding genes. In contrast to the new AMPs, the precursor protein of the previously studied rip-thanatin is characterized by the presence of 2 ÷ 4 tandem repeats and a total length ranging from 102 to 148 aa [13]. Thus, all four peptides (rip-thanatin, Rip-2, Rip-3, and Rip-4) are united only by the common negative charge (at neutral pH) of the prodomain. 

### 3.2. Production and Structural Analysis of the Discovered Peptides

**Figure 2:**
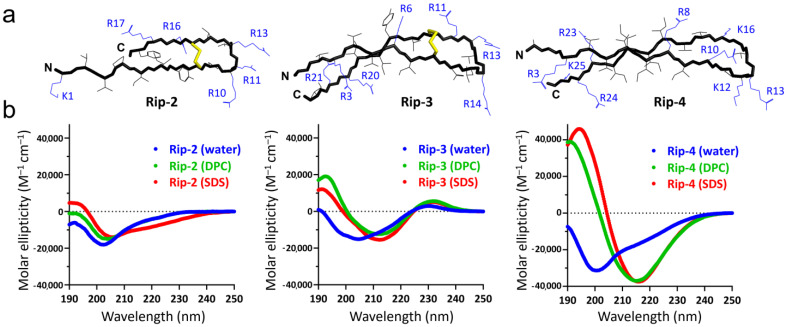
Structural analysis of the discovered peptides. (**a**) Spatial structures of Rip-2, Rip-3, and Rip-4 were modelled using the AlphaFold3 algorithm (https://alphafoldserver.com/, last accessed 11 September 2024) with default parameters. The top-rated predicted models were visualized by the PyMOL program. (**b**) CD-spectra of the discovered peptides in water (blue), or in the presence of DPC (green) or SDS (red) micelles.

The peptides were obtained as part of the fusion proteins containing the His tag and modified thioredoxin A [M37L]. After cleavage of the fusion proteins with CNBr, the corresponding mature AMPs were purified by RP-HPLC to reach a purity of ≥98%. Final yields were at least 10 mg per 1 L of bacterial culture for each peptide. All peptides under study were predicted to form β-hairpin structures by the AlphaFold algorithm [14] (Figure 2a). The predictions were confirmed by circular dichroism spectroscopy (CD) analysis in aqueous solutions and in micelles mimicking negatively charged (SDS) and zwitterionic (DPC) membranes (Figure 2b). The structure of Rip-2 is generally similar to that of thanatin, with an antiparallel β-sheet structure from Ile5 to the *C*-terminus. This is also supported by the presence of identical spectra for thanatin and Rip-2 [15]. The spectrum of Rip-3 in water suggested the presence of a flexible chain, while an increase in the molar ellipticity of the band at ~190 nm and a small red shift from 205 nm to 215 nm indicated the formation of a more rigid and ordered β-hairpin structure in the micelles. The CD spectrum of Rip-4 in water showed a negative peak at ~200 nm (Figure 2b), indicating that the peptide has mainly the random coil conformation [16]. Peptides adopting a β-sheet conformation have a characteristic CD spectrum, with a positive band at ~195 nm and a negative band at ~217 nm [16]. Similar CD bands were observed for Rip-4 (Figure 2b) in the membrane-mimicking environments, thus confirming the predicted fold. Despite the overall similarity in spatial structure (Figure 1b), the degree of homology of Rip-3 and Rip-4 with known AMPs did not exceed 45%, allowing us to identify them as new subfamilies of β-hairpin AMPs (Figure 1c).

### 3.3. Rip-3 and Rip-4 Have Different Mechanisms of Action and Spectra of Activities from Thanatin

**Figure 3:**
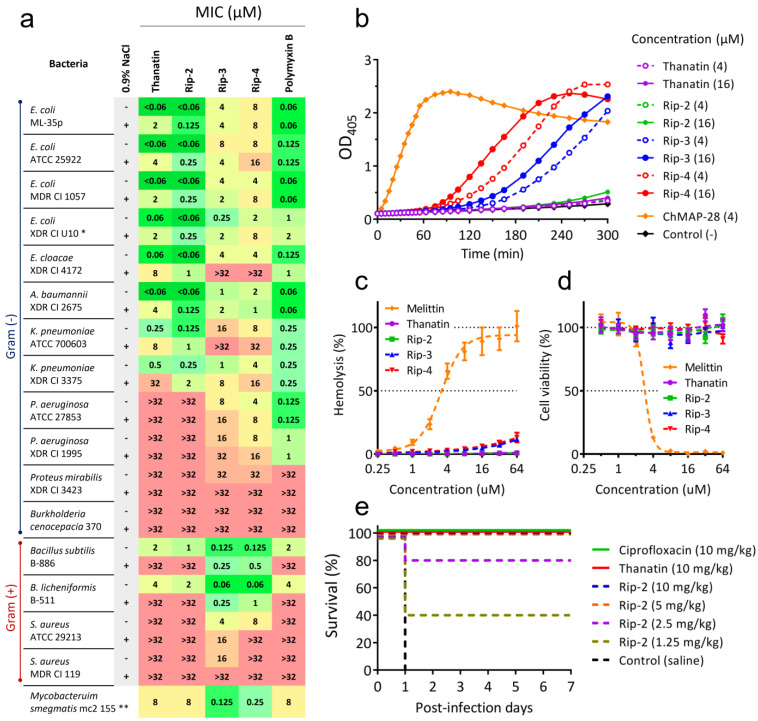
Therapeutic potential and mechanisms of action of new thanatin-like peptides. (**a**) Antibacterial activities of the peptides in comparison with those of thanatin and polymyxin B. Minimum inhibitory concentrations (MICs) were determined in rich MHB medium ± 0.9% NaCl. *—A strain with MCR-1-mediated colistin resistance; **—MICs were determined in 7H9 Middlebrook broth. (**b**) Kinetics of changes in *E. coli* ML35p cytoplasmic membrane permeability caused by the peptides (ONPG-assay). (**c**) Hemolytic activity of the peptides after 2 h incubation (the hemoglobin release assay). (**d**) Cytotoxicity of the peptides against PBMC cells after 24 h incubation (the resazurin assay). (**e**) Survival rates of BALB/c mice (n = 5) infected IP with *E. coli* ATCC 25922. The high-level CFU burden was confirmed in animals from the negative control group.

Antibacterial activities of the discovered peptides were evaluated by the broth microdilution assay against a broad panel of test strains, including multidrug- and extensively drug-resistant (MDR and XDR, respectively) clinical isolates (Figure 3a). Screening for antibacterial activities revealed significant differences in the specificity of action of the new AMPs: While Rip-2 was generally characterized by the spectrum of activities of thanatin, Rip-3 and Rip-4 showed some broadening of their spectra against various bacteria, including Gram-positive strains, and a pronounced selectivity against *Bacillus* (MIC ≥ 0.06 μM) and *Mycobacterium* spp. (MIC ≥ 0.125 μM). Interestingly, several species of bacilli like *Bacillus thuringiensis* are known pathogens of Heteroptera insects [17]. This fact may indicate a potential biological role for the discovered peptides. 

Dissimilarity in the key mechanisms of action of the Rip-3 and Rip-4 peptides may account for such significant differences in the spectra of their activities. Thus, in contrast to thanatin and Rip-2, the peptides Rip-3 and Rip-4 effectively damaged the *E. coli* cytoplasmic membrane at concentrations near their MICs (Figure 3b), although much more slowly than α-helical membranolytic AMPs such as caprine cathelicidin ChMAP-28 [11]. All previously discovered AMPs from *R. pedestris* were unable to inhibit the growth of cultured Gram-negative bacteria of the *Burkholderia* genus, which is normally sensitive gut symbiont of the insect [4]. At the same time, unidentified host factors from the M4 midgut may alter the membrane integrity of symbiotic *Burkholderia* and potentiate the activity of riptocin [4]. Here, we found no activity of the discovered peptides against *Burkholderia cenocepacia* (Figure 3a). Nevertheless, given the broad-spectrum activity of the Rip-3 and Rip-4 peptides and their ability to damage bacterial membranes, there could be speculation about their possible role in controlling both pathogens and other symbionts of *R. pedestris*. 

Data obtained also indicate a low cytotoxicity of the discovered AMPs against normal human cells such as erythrocytes (Figure 3c) and PBMCs (Figure 3d) at concentrations up to 64 μM, which is significantly higher than that of the MICs against susceptible microorganisms. Notably, the activity of Rip-2 against several ESKAPE pathogens (*E. coli*, *Enterobacter cloacae*, *Klebsiella pneumoniae*, *Acinetobacter baumanii*) is an order of magnitude higher than that of thanatin (Figure 3a) and is comparable to the potency of recently developed thanatin-based peptidomimetics [9]. Notably, the peptide showed similar activities against all tested strains of *E. coli*, including strain U10 with MCR-1-mediated colistin resistance [2,18]. In addition, Rip-2 demonstrated its high efficiency in a lethal model of septicemia caused by *E. coli* in mice: At a dose of 2.5 mg/kg administered twice, the peptide promoted survival in 80% of animals studied (Figure 3e). Thus, each of the discovered peptides has the potential to become a template for the development of a selective drug candidate. 

### 3.4. Structural Determinants of E. coli Resistance to the Discovered Peptides

**Figure 4:**
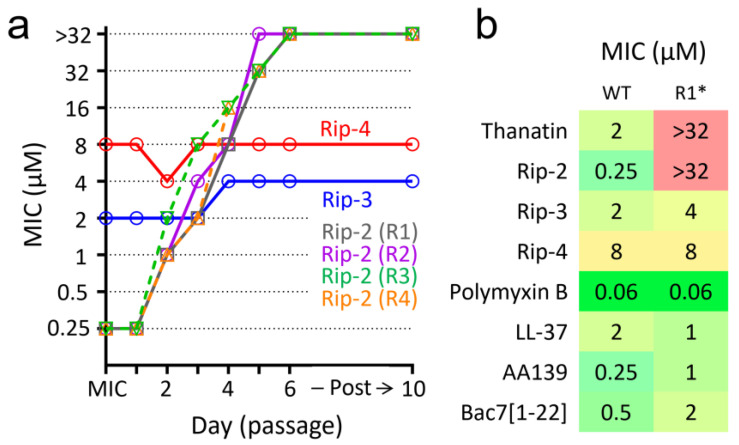
Bacterial resistance to the discovered peptides. (**a**) Serial passage resistance induction by Rip-2 (four independent experiments), Rip-3, and Rip-4 against the sensitive clinical isolate *E. coli* MDR 1057. (**b**) Evaluation of cross-resistance effects for the R1 strain. An antibacterial activity was determined in rich MHB supplemented with 0.9% NaCl. *—The whole-genome sequencing of the R1 strain showed the point mutation in the *lptA* gene [GAA→GAC; E84D] and the nonsense point mutation in the *wecA* gene [CAG→TAG; Q293stop].

A selection of resistant bacteria provides insight into both key targets of antimicrobial agents and mechanisms of protection. Repeated daily broth microdilution tests were used to induce resistance of *E. coli* MDR 1057 to the discovered peptides (Figure 4a). While an ability of Rip-3 and Rip-4 to bind to specific targets on bacterial membranes or inside the cell cannot be excluded, these molecules showed a low tendency to induce bacterial resistance in vitro, which, together with the data on the membrane permeability, suggests a membrane-targeting mechanism of their action.

On the contrary, an 8- to 32-fold increase in MICs was observed after three passages of the bacteria selected by using Rip-2 (Figure 4a). Analysis of mutations in the *lptA* and homologous *N*-terminal parts of the *lptD* genes in four bacterial strains (R1-R4) obtained after three passages showed that two of them had point mutations leading to the following changes: LptA[E84D] in the R1 strain and LptA[D41G] in the R2 strain. These residues have previously been described as key ones for thanatin and its modified analogs when interacting with the LptA protein [7,9]. An additional three passages (a total of 6 days of the experiment) resulted in an increase in the MIC > 32 μM in all cases. To determine mutations which further contribute to the increase in the MIC of the R1 strain from 4 μM (three passages) to >32 μM (six passages), whole genome sequencing of this strain was performed. Thus, in addition to the LptA[E84D] mutation, a nonsense point mutation in the *wecA* gene (Q293stop) was identified in this resistant strain. WecA is the membrane protein that initiates the biosynthesis of O-antigen of LPS by catalyzing the transfer of N-acetylglucosamine (GlcNAc) 1-phosphate to the undecaprenyl phosphate lipid carrier [19]. The mutation discovered probably results in biosynthesis of a truncated version of the enzyme (293 of 367 residues), which lacks several *C*-terminal transmembrane helices [19]. Notably, the absence of O-antigen in the cell envelope and overall compromised membrane integrity were responsible for the increased susceptibility of the *Burkholderia* bacterial symbionts to molecular factors of the *R. pedestris* innate immunity, particularly to its AMPs [4]. Here, we found that the Δ*wecA* strain from the KEIO collection did not differ from the wild-type *E. coli* BW25113 in sensitivity to the Rip-2 peptide and thanatin (the MICs of 0.25 and 4 µM, respectively). Thus, the detected mutation probably does not lead to the inactivation of the enzyme, but modulates its activity, which contributes to the structure of the cell envelope and affects activities of different cationic AMPs (Figure 4b, Table S2). A potential dependence of the Rip-2 activity on LPS structure is not surprising, as a number of studies have shown a high affinity of thanatin for this key component of the outer membrane [8]. Taken together, these findings indicate that thanatin and Rip-2 have similar molecular targets and mechanisms of action.

## 4. Conclusions

The principles for predicting the biological activity of β-hairpin AMPs are still unclear, despite the growing number of structure-activity relationship (SAR) studies of these molecules. An alternative approach to the development of promising antibiotic prototypes is the search and screening of novel families of animal AMPs selected by nature. To date, RNAseq data for >300 Heteroptera species have been deposited in the Sequence Read Archive (SRA) of the NCBI database, opening the way to identify novel families of β-hairpin AMPs such as thanatin variants with improved antibacterial activity. As proof of concept, here we discovered new subfamilies of thanatin-like β-hairpin AMPs in the genome and transcriptome of the bean bug *Riptortus pedestris*. These new peptides are likely to be universal host defense factors, as homologous genes were found in different members of the infraorder Pentatomomorpha by bioinformatic searches in the TSA and WGS databases (Figure 1c). Rip-2 has the most similar structure and LptA-targeting mechanism of action to those of thanatin, but possesses a higher activity against key Gram-negative ESKAPE pathogens. The Rip-3 and Rip-4 peptides have a different mechanism action compared to that of Rip-2 and are characterized by a pronounced selectivity against *Bacillus* and *Mycobacterium* spp. Preliminary data on the efficacy and safety of the new disulfide-stabilized β-hairpin peptides Rip-2 and Rip-3 point to their therapeutic potential for development of selective antibiotic drugs.

## Data Availability

The original contributions presented in the study are included in the article/supplementary material, further inquiries can be directed to the corresponding author.

## Supplementary Materials

The following supporting information can be downloaded at https://www.mdpi.com/article/10.3390/pharmaceutics16111453/s1, Figure S1: Analysis of Rip genes found in *R. pedestris*; Table S1: Oligonucleotide primers used in this study; Table S2: Amino acid sequences and molecular masses of the peptides used in this study. References [11,12,20,21,22] are cited in the supplementary materials.
