# Supplementary material for: Discovery of Novel Thanatin-like Antimicrobial Peptides from Bean Bug Riptortus pedestris
Source: Pharmaceutics. 2024 Nov 14;16(11):1453. doi: 10.3390/pharmaceutics16111453 (PMC11597323; doi:10.3390/pharmaceutics16111453)
Supplement: Supplementary file 1 [file pharmaceutics-16-01453-s001.zip › pharmaceutics-3289831-supplementary.pdf]

Supplementary Materials

Discovery of Novel Thanatin-Like Antimicrobial Peptides from Bean Bug *Riptortus pedestris*

Pavel V. Panteleev [1,2,*], Julia S. Teplovodskaya [1,2], Anastasia D. Utkina [1,2], Anastasia A. Smolina [1], Roman N. Kruglikov [1], Victoria N. Safronova [1], Ilia A. Bolosov [1], Olga V. Korobova [3], Alexander I. Borzilov [3] and Tatiana V. Ovchinnikova [1,2]

[1] M.M. Shemyakin & Yu.A. Ovchinnikov Institute of Bioorganic Chemistry, Russian Academy of Sciences, 117997 Moscow, Russia; juliyateplovodskaya@gmail.com (J.S.T.); nastin-u@yandex.ru (A.D.U.); a.a.smolina003@mail.ru (A.A.S.); kruglikov1911@mail.ru (R.N.K.); victoria.saf@ibch.ru (V.N.S.); bolosov@ibch.ru (I.A.B.); ovch@ibch.ru (T.V.O.);

[2] Moscow Center for Advanced Studies, 123592 Moscow, Russia

[3] State Research Center for Applied Microbiology & Biotechnology (SRCAMB), 142279 Obolensk, Russia; korobova@obolensk.org (O.V.K.); borzilov@obolensk.org (A.I.B.)

* Correspondence: p.v.panteleev@gmail.com

Rip-2

GenBank: GKOW01048511.1 (transcriptome data)

atgactactacaaacatcgtcttacttttggtctgtctttcactagtttgcatctcgctagcacaggagtcacaaccagaacaaccaaataatctgagaagagcgccgagattattttttccatgctacttccctcctttctttcgtgggcctagattactgaacgacgattcctcaatcaatgaagaggaaccctatgaaattattgaaaatgagagatcc
accgatgaaggagaagatggtccaaaaggaactgatcacccagcagacgaattggtgagaaacaaaaggaaggttgtaccaataatttattgcaacaggaggacaagagtttgtaggcgtttttag
MTTTNIVLLLVCLSLVCISLAQESSQPEQPNNLRRAPRLFFPCYFPPPFFRGPRLLNDDSSINEEEPYEIIENERSTDEGEDGPKGTDHPADELVRNKRKVVPIIYCNRRTRVCRRF

GenBank: JADPXZ010000002.1 (genome data)

atgactactacaaacatcgtcttacttttggtctgtctttcactagtttgcatctcgctagcacaggagtcacaaccagaacaaccaaataatctgagaagagcgccgagattattttttccatgctacttccctcctttctttgtaagtatcatacaaatattcaaacacacgacaagcacaaaaaacatacatttatatattttaaataatacattcctgt
ggggaaacaaaggcattactatttcattactcgaagtttgagctgcctactgaaggcccaccgtgacaggataaacataaaaaacattttctactaacgattttgtaataatagttttgatgtatttacaaatggacgttgtcatacagaaatattgatgcacgacagtgcattggagacaaactggtcctacgttttaaagtagcttgggcttgtcactta
ctccatgaaaacttaaaataataatatatatatgtatatatatatatatatatatatatatatatatatatatatatatatagatgctgatgataggatttaattttttttcatctttcatatattatgaagtcaatgaatatttagaaattgttttagaattcattcatatgcataatctaaataatttcttcatgggaattctgctataaaatgca
tttttcccagggtcatttcctttgtacgaagcctaaatattttcagtactattccttctaatactgataagtatattttggttgctggaagttatgaatgtaaaaactatttcataagaacttttagtactgaaaaatacatttttaatgaaccttttttgaatttatgtaacttagtacgttgtaactagtaataggtgacattttttgaatattgaaactg
tttttaatggtggggcgtccgagagaataattatgtacaaaaactcaagaattcaacactcaacaattagcttgaaaaatgagtgttatcatattaagtaagataattgttgaaagtatagaatacatactaaaatcatattgattattgaactaagccaatcaattacaatttaaaatcaattgtctgtatttaaaaaataaatacaagtaaaaaattt
tagaagctaagaaaattaatttttttcagcgtgggcctagattactgaacgacgattcctcaatcaatgaagaggaggaaccctatgaaattattgaagatgaggtatgtttggatgcacttaaaaagttaatacataaaaatgacttcaaaaaatattaacaatttaattataaatcctaatgaaggatgttttttatgcagccacagagatccactgat
gaaggagaagatggtccaaaagaaactgatcacccagcagacgaagtatgttacattacatttatatagagaatcgaacatattattatttagtttcactaaatcattagaaggaaaaaaggtagttgatctattttgaacaaactcaattaacgataaactatcacgaaatgcactccatgttttaaaactatgcggatacatttttagcagatttccctt
aagggaaatcagctattgaggtcgtactcatgatgcgagtttcgaaaaatttcttcaaatatccatcgatcttacctaggagatgacgaaaatgaggtccgttcaaaattatatatataatatatatatatatatatatatatatatatatttgtttgttaacgctctagcggtcgcaattattgtttgatcgttaccaaactcggaatttaatcgtttt
atgtaaaaaggaagatcaagttcgttaacggctgaaatcagaccagtgggaggggttgagaggggagttttgaaaattcaaaatatctccttttattatgcgtcggatcgaaaaaatagtatagaataaatcttattgcaaattaaatttactataaatattgttctatacatttattctgtatcttgaatactaaccgagatatttgcatttgtattctgcgt
gtggtcaccagtaggctatgttcggactttccgacgagagctctggtggccgtttcccataactattcgtctctcgtgaataggtttagaggttaggttaactgtttttcagggctagaaactacagggttattaccccctaaataatgttttaggtttagaggtttcttactactacagttgtgattaggcagatttctctattgtaaaacttcacgtggga
aatctgcttagtttacgcagcccctgcgtgatggactttctatttccgatgcctttgtcgtttgatagactcaagcaggagacaacgggaacctctaaatattctactactgtctctcttctttcttcctgtggacagaagtacatactgtccacatataacaaatacggcttcaaatggaactcttaagaataaataagaaaaatatttcatgagaattcaa
atattttgttgatggccaaccttatttaatgcaatctcctcctggagaaatagttagatcgaatactaatgttttacatacttaaacaaaatatcactatacatttagttcttttatgttaaaatggttcaatattaacacaattgtatgtatattcttgttacagttggtgagaaacaaaaggaaggttgtaccaataatttattgcaacaggaggacaag
agtttgcaggcgttttttag
MTTTNIVLLLVCLSLVCISLAQESSQPEQPNNLRRAPRLFFPCYFPPPFFRGPRLLNDDSSINEEEEPYEIIEDERSTDEGEDGPKETDHPADELVRNKRKVVPIIYCNRRTRVCRRF

Rip-3

GenBank: GKOW01018728.1 (transcriptome data)

atggcttcctggatgacgttccttcttctggcattcgttgctctggccagccttgcttcagctcagagatacacgtcgcctctcaaacaaccggccgtaccaagatacattaaccgaccaaaaccacctccttcatttggaaacagaaggttcagaagatcagcaaccgaggaagaaatagtacacaacaacagtgagtggacagtcgaagagatagtagat
gaaaaggatctgcctaaggccggagataacgttgtatatgaaggagtacaggatgaagtcttaaaatccgagggcgagaagagtcgaagcaaaagagctgttcgagtgacaagaatttgcaacttacgaacaaggagatgtgtttatataatcagaagaatatag
MASWMTFLLLAFVALASLASAQRYTSPLKQPAVPRYINRPKPPPSFGNRRFRRSATEEEIVHNNSEWTVEEIVDEKDLPKAGDNVVYEGVQDEVLKSEGEKSRSKRAVRVTRICNLRTRRCVYIIRRI

GenBank: JADPXZ010000002.1 (genome data)

atggcttcctggatgacgtttcttcttctggctttcgttgccctggccagccttgcttcagctcagagatacacgtcgcctgtcaaacaaccggccgtaccaagatacattaaccgaccaaaaccacctccttcatttggaaacagggtaagtgcaaaatgaaactatctttcaactaatgaggttaaaataaaaatatggcgtcagcaccacatggctccc
ttttattctaattaaattaaacgtataaaaattgcgttgcacatttcttgaaattatttcaatcggaagtcatatcctatatctccgttccaattcatcgggttgaaaaatttccatagccacctccacgcggttcatgatcttttttatcacaaaaaaaaaagtcatatacaaatattttgtaactctcataggtacagagaaattggaaatttttattttc
acccccgagactccctccctttctcaaaatttgtctattttgatcttaaggagaagtggacatcaaaaattgtcaacaatcctaaatatgaaatttcaccattctaagactagccaaaaagttaaatttcctaggaaatatgaaactattaattttttttttgttgcaatcacaacttttttcattcacttcttacgaggaagtaaaaagtccgccaagcaggg
gctgcgtcaaataagcagatttcccacttgtgtcacgtaacctaatttacttcaaaacttagaagctcaatgaaaacaatatatggtacttaagcttctaagaaaatgcttcataatataactgattttttgttaatatgatattagtaggagtcgggggttactgatagctaaagtatggaaatatctcggttaatattcaatttagagaaaatatgttta
tagcaacatatatagaaaatcgcatttgcaatagatgattgattctattaaatgttttgatccgaatttacaaaattctcctcctctaacccccccccccccaccactggtccgaatttaaccctaaggaacttgatcttcatttttatattaaatgtttaactatcgagtttggtaacgataagaaaataattgcgagcaatagagctttaacagggatatat
at
gtttaatccagaaatatagaaatttacaaccatttcctccaaaatcaaaacatccaaatttgaaacgtttggactcaaaggttttgaaagctgtctggcgatgtgtaaggattatccctcactcaataaaactaaaaattggcaggagtataaaaatcaacctaactaaattatgttgaatttacagaggttcagaagatcagcaaccgaggaagaaatagt
acacaacaacagtgagtggacagccgaagagatagtagatgaaaaggtaaacacagttcaaaacatatttattcatttatgtatatatatatatatatttgtgtgtattcaatagtatatttaagcattacaataaatgaattaccatttttaggatctgcctaaggccggagataacgttgtatatgaaggagtacaggatgaagtcttaaaatccgagggc
gaggtaagaaaaacattgaattggtttactttgtttgatttttaaaaacgatttttctggacgatttggaaatgctatatacttattatttaagcatatttcccacttgttggtcggaccacacgccatataatactgtgctctgatttctttcttagttaagaccgtatgtaaacaagccttggtatttatgttgtacctaagatattatgaaaaaaaaaagt
ccgtcacacactaactgcgtaaactaagcagatttcccacgcgaagttatacaatagagaaatctccgaatgagagccgaacagttacggccgaactagcttttggcgggaacctcactcgtccgttgtcggcccacatctaataaaatgcaaatatctcggttaataatcaacatagagaagaaatgtatagaagaatatttctagtaaatataatttgc
aataagattgtttttttactatttttgatccgacgtatggaaacggagatatttcgaatttttgaaaatccctcccctaaacccaccctcccactggtccaataacaaaaattgcgaccgctagagcgttaacagacatgcatacacacacacacacacacacacacacacacacacacacacacacacacacacacacacacacacatatatatatatat
atatatatatatatatatatatatatatatatatatatatacatatatatacatatacatacatatacatataatttttgaacggacctcattttccgatcgatggacatttgaagacatttttcgaaaatcgcgtcatgagttcgacctcaatagctgatttcccttagtgaaaatctgctaatagagtggaggcggaagtatatatctacaaagtgctcgataaatttg
gcgtagacaataatcactctaaaaatgattggcagaacttattctctcactattacttactctcttactatacatatttttaaccgaggataaaccgaggttaaataacggatttgttatattttcatatcaataattttacatgtaaagtatatttgatagtgatataataggtaatatggaatcacatttgatcaagtatttaaattacgttaaaata
aggaaatgcatgtaccctcaagttaaaaattaaaaataccatacggaacattatggtcttataaagtaatacaataatacaataggatataaaattctttgatggatattttatactctatattttgtagtggcctttgagaaattagtatttcatcttgttcaattgaaaatccatgcaatagaatctaacaaacgttattataaaattaagaaatcatt
aatcaaaataatattttttcagaagagtcgaagcaaaagagctgttcgagtgacaagaatttgcaacttacgaacaaggagatgtgtttatataatcagaagaatatag
MASWMTFLLLAFVALASLASAQRYTSPVKQPAVPRYINRPKPPPSFGNRRFRRSATEEEIVHNNSEWTAEEIVDEKDLPKAGDNVVYEGVQDEVLKSEGEKSRSKRAVRVTRICNLRTRRCVYIIRRI

Rip-4

GenBank: GKOW01051453.1 (transcriptome data)

atgtctatggagaagatatttgtacttgtagccactttggctatctgcagcacttctttgggacaagcctttgcaccctggggcccaagaagagatgaaggacttcaattgccatttcgaaggggtcctcttgatccttgggtgcaacgcagagaaccacctccttttgttcgtcccgtcagatcggtccaggaagacaactacccaatagcagaagatctt
tacgaagatgtatccaatgatgagcggttcaagagagcaacaaatgaacaattggacgctacaaatgaatggacagaaggagaaatcattgaggaaaaggatattccaaagggagctgagatcattgaagtgtcgaaggatggtgagtttgaagaaatcacagaggaaaaatcacctgttgcgaggagtaaacgagctgcgcgcgtaactataataaggatc
aggaacaagagaacaggaaaagttaccataatcgttataagaaggaaatga
MSMEKIFVLVATLAICSTSLGQAFAPWGPRRDEGLQLPFRRGPLDPWVQRREPPPFVRPVRSVQEDNYPIAEDLYEDVSNDERFKRATNEQLDATNEWTEGEIIEEKDIPKGAEIIEVSKDGEFEEITEEKSPVARSKRAARVTIIRIRNKRTGKVTIIVIRRK

GenBank: JADPXZ010000002.1 (genome data)

atgtctatggagaagatatttgtacttgtagccactttggctatctgtagtaattccttgggacaagcctttgcaccctggagcccaagaagagatgaaggacttcaattaccatttcgaagaggtcctcttgatccttgggtgcaacgcagagaaccacctccttttgttgtaagtcacttcaaggcgtttacgaatagcaaagctaaatatcatttttttt
aaaaatttcatcgcttaagtggcgtttcacaaataagcaacttatttttaaagtagaaattagaacgcataaagcaactgttacatccatgagacggaaataaaatggaaattttacctcagtttcagatctactgatattgaaaaacgtccgtcacgcaggagcatacgttaactaagcagatttttcgacgtgcgtcacgtatttgcatttatctcaaaact
taaaaattcaatgagtaccatatttggtattcagaattcaaataatgtgctacaaaatataacagttcatcgctaaattaaaatatctgttttaataatcaagataatgaaaaaacgtacataataacaagtatagagcattaaacggtctataagatggttctatgcaatttttcggtccgacgcatagtaacgcagatattcagaatattcaaaatttc
tctctctaacccccctccctcccccttggtcgtatttcagccattaacgaacttgatctatagttttacattaaaaatttaaatatcaagcgtaggtttgaatctcactttgcacgcctagagaagtgggttcgactcacccgcatggacaataggaatttatttagatatgaactaatgaaataaaatccttagtcccacggccatggctagaaatgggtaat
atcagctaaatcaaccgtccaaattagccaaagtagcacaaagtgaatgttattaaacaacattaacgatagcgtttaatataacattcacaagcataactttataaaccaactcctccctttcacccatgtacctacatatgacatatatacgcacgtgtacacttacacatttatatgtacctaatttcatttagtggcatcaaattgaaaagtcatct
tagtcgcgaaaagttgaaaactgtctccacttgtttttaggactagttattaagaaaccatttctcatatttatatacaattattagtatttattatattagcaatattagtaacataattgaagagataattctacatatttattctttcagcgtcccgtcagatcggtcaacgaagacaactacccaatagcagaagatctttacgaagaattatccaat
gatgaggtagcttatacgacatattaatgataaattgtattaaaaatatatatttgttgaaataaatgaattaaatttaatataaatttcaacggtctaaattgcatactttttttttttcttgttttcagcggttcaagagagcaacaaatgaacaattggacgctacaaatgaatggacagaaggagaaatcattgaggaaaaggtacccaatattagaat
atctggagagttatttatgaaatttaccacgaatttcattgttattcccatataaagtgaaacaaaaattaatattataagttaagttcttcacttgctaaaatagtgtcaagagaattaggggtttagaggaagaaaaacgagaatcgtaaatatcaattgttatccgtcgcatgaaaaaatattgtatatgtaatcttgtttttcttctatggttttgttttc
tatggtatcttctattttcttcaattatgaggtacaatttttttctgaaacccgaacattagtggagttatttgtaatttatatttcttaatagaaaatcataagcaggtgactttctcattaaactgcctatacactgtatgcatgtggctaaatttcaattcttgtttacaaactattctcactaagggagaaatttagaatagacagtaagatgatg
tcagctttaagtcattccagcaggaagaaaatctttagctgaccccctgcataactttcttcttgaagttgaaaataacatgtattactagaacttccctactcaccctaatacacagcctgtgtgagaggtttttgttgtgaatttacttgtatttttaagagtattacaataatattcagcttcatatatattgtatgggctaaatcaactagattaaaac
attacataaaaaaaaaatcagtaataaatgaaatatatatataaattatttattactaagaacattctcttccaacaatactcctcgtatataataatgtatattttacaggatattccaaagggagctgagatcattgaagtgtcgaaggatggtgagtttgaagaaatcacagaggaaaaatcaccggtaagtacataatgtccatgatatattctgcaa
aatactctatgaaaatagcagtatccagaaggaaaaataacgtgctccttttactgtttcaggttgcgaggagtaaacgagctgcgcgcgtaactataataaggatcaggaacaagagaacaggaaaagttaccataatcgttataagaaggaaatga
MSMEKIFVLVATLAICSNSLGQAFAPWSPRRDEGLQLPFRRGPLDPWVQRREPPPFVRPVRSVNEDNYPIAEDLYEELSNDERFKRATNEQLDATNEWTEGEIIEEKDIPKGAEIIEVSKDGEFEEITEEKSPVARSKRAARVTIIRIRNKRTGKVTIIVIRRK

Figure S1. Analysis of Rip genes found in *R. pedestris*. Exons are highlighted in green; introns are highlighted in light grey. Signal peptide sequences are marked in yellow/black. Differences in structures of precursor proteins are highlighted in purple.

Supplementary Table S1. Oligonucleotide primers used in this study.

Name	Sequence 5'→3'	Description
Rip-2f	CGAGATCTATGAAGGTCGTTCCAATTAT CTACTGCAACCGGCGGACA	Synthesis of fragment encoding Rip-2 followed by insertion into pET expression plasmid
Rip-2r	GCGAATTCTTAAAAGCGACGACAAACCC GTGTCCGCCGGTTGCAGTAGA	
Rip-3f	CGAGATCTATGGCCGTCCGCGTCACCCGT ATCTGCAACCTTCGTACCCGCCGTTG	Synthesis of fragment encoding Rip-3 followed by insertion into pET expression plasmid
Rip-3r	GCGAATTCTTAGATACGACGAATGATGTA AACGCAACGGCGGGTACGAAGGTTGC	
Rip-4f	CGAGATCTATGGCAGCCCGCGTGACCATTA TCCGCATTCGCAACAAGCGCACCGGCAAA	Synthesis of fragment encoding Rip-4 followed by insertion into pET expression plasmid
Rip-4r	GCGAATTCTTATTTGCGACGAATCACAATG ATAGTAACTTTGCCGGTGCGCTTGTTGCG	
LptA-f	GCCGAGCTGATTGAAAAGGT	Amplification of lptA gene of *E. coli* (TA-cloning and sequencing)
LptA-r	ACGCTGAGGCGACGGAAAAT	
LptD-f	GAAAAAACGTATCCCCACTCTC	Amplification of lptD gene fragment of *E. coli* (TA-cloning and sequencing)
LptD-r	TGGCGCGATGTTCCAGTAAT	

Supplementary Table S2. Amino acid sequences and molecular masses of the peptides used in this study.

Peptide	Origin	Sequence	Molecular Mass, Da		Ref.
			Calculated $[M+H]^+$ value[1]	Measured value[2]	
Thanatin*	Rec	GSKKPVPIIYCNRRTGKCQRL	2415.32	2415.36	[1]
Rip-2	Rec	KVVPIIYCNRRTRVCRRF	2277.28	2277.14	This study
Rip-3	Rec	AVRVTRICNLRTRRCVYIIRRI	2728.60	2728.16	
Rip-4	Rec	AARVTIIRIRNKRTGKVTIIVIRRK	2931.89	2931.24	
AA139	Rec	GFCWYVCARRNGARVCYRRCN	2549.17	2549.95	[2]
Bac7[1-22]	Rec	RRIRPRPPRLPRPRPRPLPFPR	2783.72	2783.89	[3]
ChMAP-28	Rec	GRFKRFRKKLKRLWHKVGPFVGPILHY	3364.00	3364.22	[4]
Melittin	Synt	GIGAVLKVLTTGLPALISWIKRKRQQ	2846.74	2846.60	[5]
LL-37	Synt	LLGDFFRKSKEKIGKEFKRIVQRIKDFL RNLVPRTES	4491.58	4491.40	[3]

* recombinant thanatin [M21L] analog

[1] according to Expasy Isotopident tool

[2] monoisotopic m/z were measured using MALDI-TOF MS (the measured m/z values match well the corresponding calculated molecular masses)

Supplementary references:

1. Panteleev, P.V.; Balandin, S.V.; Ovchinnikova, T.V. Effect of Arenicins and Other β-Hairpin Antimicrobial Peptides on Pseudomonas Aeruginosa PAO1 Biofilms. *Pharm Chem J* **2017**, *50*, 715–720, doi:10.1007/s11094-017-1518-2.
2. Krenev, I.A.; Panteleev, P.V.; Umnyakova, E.S.; Gorbunov, N.P.; Kostevich, V.A.; Balandin, S.V.; Ovchinnikova, T.V.; Aleshina, G.M.; Berlov, M.N. In Vitro Modulation of Complement Activation by Therapeutically Prospective Analogues of the Marine Polychaeta Arenicin Peptides. *Marine Drugs* **2022**, *20*, 612, doi:10.3390/md20100612.
3. Panteleev, P.V.; Safronova, V.N.; Kruglikov, R.N.; Bolosov, I.A.; Bogdanov, I.V.; Ovchinnikova, T.V. A Novel Proline-Rich Cathelicidin from the Alpaca Vicugna Pacos with Potency to Combat Antibiotic-Resistant Bacteria: Mechanism of Action and the Functional Role of the C-Terminal Region. *Membranes* **2022**, *12*, 515, doi:10.3390/membranes12050515.
4. Panteleev, P.V.; Bolosov, I.A.; Kalashnikov, A.À.; Kokryakov, V.N.; Shamova, O.V.; Emelianova, A.A.; Balandin, S.V.; Ovchinnikova, T.V. Combined Antibacterial Effects of Goat Cathelicidins With Different Mechanisms of Action. *Front. Microbiol.* **2018**, *9*, 2983, doi:10.3389/fmicb.2018.02983.
5. Panteleev, P.V.; Myshkin, M.Yu.; Shenkarev, Z.O.; Ovchinnikova, T.V. Dimerization of the Antimicrobial Peptide Arenicin Plays a Key Role in the Cytotoxicity but Not in the Antibacterial Activity. *Biochemical and Biophysical Research Communications* **2017**, *482*, 1320–1326, doi:10.1016/j.bbrc.2016.12.035.
